# Candidate gene study of macular response to supplemental lutein and zeaxanthin^☆^

**DOI:** 10.1016/j.exer.2013.07.020

**Published:** 2013-10

**Authors:** Ekaterina Yonova-Doing, Pirro G. Hysi, Cristina Venturini, Katie M. Williams, Abhishek Nag, Stephen Beatty, S.H. Melissa Liew, Clare E. Gilbert, Christopher J. Hammond

**Affiliations:** a Department of Twin Research and Genetic Epidemiology, King's College London, UK; b Institute of Ophthalmology, University College London, UK; c Macular Pigment Research Group, Waterford Institute of Technology, Waterford, Ireland; d Novartis Pharmaceuticals Corporation, USA; e International Centre for Eye Health, London School of Hygiene and Tropical Medicine, UK

**Keywords:** macular pigment, lutein, genetics, supplementation, macular degeneration, CAREDS, Carotenoids in Age Related Eye Disease Study, MP, macular pigment, L, lutein, MPOD, macular pigment optical density, LZ, lutein and zeaxanthin, Z, zeaxanthin

## Abstract

Supplementation with carotenoids is proposed to protect against age-related macular degeneration. There is, however, considerable variability in retinal macular pigment response, which may be due to underlying genetic variation. The purpose of this study was to determine whether genetic factors, which have been previously associated with cross-sectional macular pigment levels in the retina or serum lutein, also influence response to supplementation.

To this end we conducted an association study in 310 subjects from the TwinsUK cohort between variants in 8 candidate genes and serum lutein and retinal macular pigment optical density (MPOD) levels before and after supplementation. Four variants were associated with MPOD response to supplementation (*p* < 0.05): rs11057841 (*SCARB1*), rs4926339 (*RPE65*), rs1929841 (*ABCA1*) and rs174534 (*FADS1*). We also confirmed previous associations between rs6564851 near *BMCO1* (*p* < 0.001) and rs11057841 within *SCARB1* (*p* = 0.01) and baseline measures of serum lutein; while the latter was also associated with MPOD response, none of the *BMCO1* variants were. Finally, there was evidence for association between variants near *RPE65* and *ELOVL2* and changes in lutein concentration after supplementation.

This study is the first to show association between genetic variants and response to carotenoids supplementation. Our findings suggest an important link between MP response and the biological processes of carotenoids transport and fatty acid metabolism.

## Highlights

• Four variants were associated with macular pigment response to supplementation.
• We replicated associations between *BMCO1* variants and lutein at baseline.
• Carotenoids transport may affect macular response to supplementation.
• Lipid metabolism may affect macular response to supplementation.

## 1 Introduction

Age-related macular degeneration (AMD) is the leading cause of blindness in developed countries. Age is the most important determinant, and common genetic polymorphisms are important in its etiology (Evans, 2001; Fritsche et al., 2013; McCarty et al., 2001). Oxidative stress has been implicated in AMD pathogenesis, together with other risk factors such as smoking, obesity and hypertension (Beatty et al., 2000; Evans, 2001; Hyman et al., 2000; Johnson, 2005; Smith et al., 2001). Macular pigment (MP) may play an important role in protecting the eye from accumulation of oxidative species by absorbing blue light and thus reducing light-induced oxidative stress, as well as having a direct antioxidant effect (Beatty et al., 2000; Margrain et al., 2004). MP represents the accumulation of the carotenoids lutein (L) and zeaxanthin (Z), as well as meso-zeaxanthin, a derivative of lutein. Lutein and zeaxanthin (LZ) are xanthophyll carotenoid pigments which cannot be synthesized *de novo* in mammals and are predominantly derived from fruits and vegetables. Therefore, carotenoids-rich diets and LZ dietary supplements have been proposed to protect against AMD (Delcourt et al., 2006), and supplements have been tested in the Age-Related Eye Disease Study 2 (AREDS2) (AREDS2, 2013). Although additional LZ supplementation to the AREDS1 formulation did not significantly reduce progression of AMD in the AREDS2 primary analysis, secondary analysis suggested that LZ supplementation was protective against AMD progression in individuals with low LZ dietary intake. Moreover, when LZ was given without β-carotene there was a statistically significant reduction in AMD progression. This, and the fact that addition of β-carotene increases the risk of lung cancer in former smokers, led the authors of the AREDS2 study to conclude that LZ could be an appropriate carotenoid substitute in the AREDS formulation (AREDS2, 2013).

The accumulation of LZ in the macula is dependent on a variety of factors – absorption, digestion, transport, retinal uptake, macular storage, degradation and secretion. However, little is known about the extent to which genetic variation influences these mechanisms. To our knowledge there are no studies on heritability of serum xanthophyll levels, but there is data on heritability of serum LZ concentration (between 67% and 85%) and on heritability of macular pigment optical density (MPOD) response to LZ supplementation (27%) (Hammond et al., 2012; Liew et al., 2005). Genetic studies, both genome-wide association studies (GWAS) and studies at a candidate gene level, have explored genetic factors that underlie variation in serum LZ and MPOD measures, with some success. There have been GWAS on plasma levels of carotenoids alone (Ferrucci et al., 2009) and on AMD and circulating carotenoid levels (Cipriani et al., 2012; Kopplin et al., 2010; Yu et al., 2011). Ferrucci et al. (2009), in a GWAS of 1190 people, found significant associations between plasma levels of the carotenoids β-carotene and lutein and SNPs mapping to *BCMO1*; the SNP rs6564851 explained 1.9% of variance. In a candidate gene study examining plasma LZ levels in 302 healthy adult subjects, McKay et al. (2013) found 5 of 47 variants in *SCARB1* associated with serum L; only one survived correction for multiple testing by permutation.

A recent study, in 1585 female participants of the Carotenoids Age Related Eye Disease Study (CAREDS), investigated associations between 440 SNPs in 26 candidate genes and MPOD (Meyers et al., 2013). Variants in 11 genes were associated with MPOD and collectively explained 5.1% of variance, though only rs11645428 in *BCMO1* survived Bonferroni correction for multiple testing.

Given that there is considerable variability in MPOD response in the (generally small) supplementation studies (Connolly et al., 2011) and that AREDS2 showed LZ supplementation to be effective in absence of β-carotene (AREDS2, 2013), understanding the pharmacogenetics of supplementation response is important to determine which groups of people might benefit most from taking supplements. Therefore, the aims of the current study were to determine whether any of 12 variants in 8 genes previously associated with MPOD or serum LZ influence MPOD and/or serum response to LZ supplementation, and to replicate these associations.

## 2 Methods

### 2.1 Subjects

We studied 310 Caucasian female twins (79 monozygotic (MZ) and 76 dizygotic (DZ) pairs), aged 20–50 years (mean: 39.72, SD: 8.40), free of ocular pathology, who were previously enrolled in a prospective, non-randomized supplement study that aimed to estimate the heritability of macular response to LZ supplementation (Hammond et al., 2012). Briefly, participants were asked to take LZ supplements with food (3 tablets per day of “Macuvite” (Springfield^®^, Oud-Beijerland, The Netherlands)) for a period of 6 months. Each tablet contained 18 mg lutein and 2.4 mg zeaxanthin. The compliance was 90% at 3 months and 82% at six months (Hammond et al., 2012). All individuals were unaware of eye research interests at the time of enrollment and provided written informed consent. The research was approved by the local ethics committee and conducted in accordance with the tenets of the Declaration of Helsinki.

### 2.2 Response measurements

Serum lutein and zeaxanthin levels were measured and analyzed separately using reverse phase high performance liquid chromatography while MPOD was measured by autofluorescence (AF). We assessed response to supplementation as change of serum L and Z levels after 3 months of supplement intake and as change in MPOD after 6 months of supplementation. These changes were expressed as a rate of change as defined by the ratio of the difference between the second and baseline measurements, divided by the baseline measurement. MPOD at baseline and MPOD response were approximately normally distributed and were not transformed, but serum L and Z and the change in their levels underwent logarithmic transformation. As L and Z were correlated (correlation coefficient of 0.61), we chose to analyze L only.

### 2.3 Genotyping data

The TwinsUK cohort, a subset of which was used in this study, was previously genotyped using Illumina 610K/Illumina 317K chips and later imputed against the CEU HapMap2 panel using IMPUTE 2 (Howie et al., 2009). All SNPs passed strict quality control (QC). Briefly, SNPs with minor allele frequency less than 5% and SNPs showing deviation from Hardy–Weinberg equilibrium (*p* < 10^−4^) or high missingness were excluded. Individuals with non-Caucasian ancestry, as detected by principal components analysis, were excluded. After imputation, poorly imputed SNPs were also excluded. The final number of SNPs after the quality control step was 2,287,998.

### 2.4 Statistical analysis

Ten of the 13 CAREDS MPOD-associated SNPs from their final multivariate model were tested (there were no data on rs6078 within *LIPC* and rs675679 within *GSTP1*; rs10744182 was excluded because of low imputation quality) (Meyers et al., 2013). SNPs in *BCMO1* overlapped with SNPs in the GWAS of serum L (Ferrucci et al., 2009), only rs6564851 was added to the analysis. Finally, the SNP most strongly associated with serum LZ in the *SCARB1* candidate gene study, rs11057841, was included (McKay et al., 2013). This resulted in 12 SNPs in 8 genes being tested for association for both MPOD and serum L response to supplementation (Table 1). Where more than one SNP was tested per gene, those SNPs were independent of one another (*R*^2^ < 0.80). Eight of the analyzed SNPs were genotyped while rs11057841, rs838879, rs10179921 and rs174534 were imputed. Age-adjusted association analyses taking zygosity into account were performed using the likelihood ratio test implemented in the Merlin software package. SNPs with *p* < 0.05 were regarded as nominally significant and SNPs with *p* < 0.0019 as significant after Bonferroni correction for multiple testing (0.05/number of SNPs × 2 where 2 is the number of independent traits tested i.e. L and MPOD).

## 3 Results

At baseline, the mean MPOD was 0.41 (SD = 0.15) density units and the mean lutein concentration in the blood was 0.121 μg/ml (SD = 0.05). The mean increase in MPOD after 6 months of supplementation was 3.7% (0.015 density units (SD = 0.06)). The lutein concentration at 3 months after supplementation had increased by a mean of 125% (0.15 μg/ml (SD = 0.05)). There was no difference in L concentration or MPOD between MZ and DZ twins at either point (*p* > 0.05).

Four of the tested variants were associated with MPOD response (Table 1). The most significantly associated variant was rs1929841 (*ABCA1*) and the G allele at this variant was associated with poorer MPOD response (*p* = 0.01). Similarly, the T allele at rs11057841 (*SCARB1*) was associated with poor MPOD after supplementation (*p* = 0.02). Two other variants, rs4926339 (*RPE65*) and rs174534 (*FADS1*) were associated with better MPOD response after supplementation (*p* = 0.04 and *p* = 0.03 respectively). Two of the tested variants were associated with change in L concentration after supplementation: rs4926339 (*RPE65*) and rs1150561 (*ELOVL2*) with *p* = 0.01 and *p* = 0.04 respectively (Table 1). Of note, rs4926339 (*RPE65*) was associated with both MPOD and L response after supplementation and with MPOD at baseline (Table 2).

At baseline, three variants were associated with MPOD: rs4926339 (*RPE65*), rs6564851 (*BCMO1*) and rs6564863 (*BCMO1*) with *p* = 0.007, *p* = 0.0018 and *p* = 0.04 respectively. Only rs6564851 survived correction for multiple testing (Table 2). Four of the tested variants were associated with baseline lutein concentration (Table 2). Three of these, rs11057841 (*SCARB1*), rs11645428 (*BCM01*) and rs6564863 (*BCM01*), were nominally associated (*p* = 0.01, *p* = 0.03, and *p* = 0.01 respectively), while rs6564851 (*BCM01*) was still significant after correction for multiple testing (*p* = 0.0001). Of note, rs6564851 and rs6564863 were associated with both MPOD and L at baseline (Table 2) while the T allele at rs11057841 (*SCARB1*) was related to higher baseline L, but poorer macular response.

## 4 Discussion

This study is the first, to our knowledge, to investigate the genetics of retinal macular pigment response to supplementation with carotenoids. In this relatively small study of 310 participants, we found some evidence that rs4926339 (*RPE65*), rs1929841 (*ABCA1*), rs174534 (*FADS1*) and rs11057841 (*SCARB1*) may influence the MPOD response to supplementation (*p* = 0.04, *p* = 0.01, *p* = 0.03 and *p* = 0.02 respectively). These associations, however, were significant only at nominal statistical level, most probably due to the modest power of our sample. We confirmed with robust statistical significance the previous association reported between the variant rs6564851 in *BMCO1* and cross-sectional measures of serum L (Ferrucci et al., 2009). We also replicated the association between *BMCO1* variants and cross-sectional MPOD (Meyers et al., 2013). However, there was no evidence that rs6564851 or other *BMCO1* variants influenced MPOD or serum L response to supplementation.

The rs11057841 (*SCARB1*) was also associated with MPOD response; we and others have previously identified its association with cross-sectional serum L levels (McKay et al., 2013). Although we did not replicate all the associations from CAREDS (Meyers et al., 2013), we did find nominal association between rs4926339 (*RPE65*) and rs6564863 (*BCMO1*) and MPOD at baseline (*p* = 0.007 and *p* = 0.04 respectively). Moreover, rs11645428 (*BCMO1*), rs838879 (*SCARB1*) and rs4379922 (*SCARB1*), and rs10179921 (*ABCG5*) had the same direction and similar magnitude of effect on cross-sectional MPOD as previously reported (Meyers et al., 2013).

For most complex traits, common variants involved in complex phenotypes have relatively low effect sizes over their respective traits (http://www.genome.gov/gwastudies/). Our study is only modestly powered to detect them, as it was small (310 subjects) and the MPOD response to LZ supplementation was weak (Hammond et al., 2012). Despite this, we not only replicated variants that were previously associated with L levels or MPOD, but also were able to detect variants associated with MPOD response to supplementation. A larger study would have the power to detect more associations and variants with smaller, but nonetheless important, effects.

Three of the *BCMO1* SNPs we tested were associated with baseline L and two of those were also associated with baseline MPOD (Table 2). Although the role of *BCMO1* in carotene metabolism is well studied, the role of this gene in xanthophyll metabolism is unknown. *BCMO1* encodes a carotenoid cleaving enzyme which converts β-carotene to retinol (Lobo et al., 2012) – a process that produces reactive oxygen species in the presence of oxidative stress (Lobo et al., 2012; Siems et al., 2002). Although retinol is essential for vision and for quenching free radicals, its metabolites can also induce reactive species (Chen et al., 1999; Klamt et al., 2003; Murata and Kawanishi, 2000). Assuming that carotenoids concentration in the macula is regulated by mechanisms similar to those in peripheral blood, rs6564851 alleles might affect the fine balance between these antioxidants and their potential harmful metabolites. Also, there might be competition in absorption, transport or clearance between β-carotene and LZ which favors β-carotene, as suggested by co-administration experiments at high doses (Kostic et al., 1995; Wang et al., 2010). We found no evidence that common *BMCO1* variants are involved in response to supplementation, however.

At another variant of interest, rs11057841 (*SCARB1*), the T allele was associated with increased L levels at baseline and also with a larger increase in MPOD after supplementation – a potential protective effect for AMD. We have previously reported our replication of the association between rs11057841 within *SCARB1* and serum L (McKay et al., 2013). Of note, in this study this variant was also associated with MPOD response, a novel finding. *SCARB1* encodes a high density lipoprotein receptor, which plays an important role in the metabolism of cholesterol and L in particular (Zerbib et al., 2009), and may be involved in the formation of drusen and basal deposits during the early stages of AMD (Curcio et al., 2011; Mullins et al., 2000).

We found some suggestive association between a variant in *ABCA1* and MPOD response to supplementation (Table 2). *ABCA1* encodes for an ATP-binding cassette which functions as a cholesterol efflux pump (Schmitz and Langmann, 2001). In chicks, recessive mutations in *ABCA1* lead to reduced MPOD due to reduced co-transport of LZ from the liver to the blood and from the blood to the retina (Connor et al., 2007). Although a high L diet could rescue the liver phenotype, the retinal LZ concentration remained low (Connor et al., 2007). In humans, *ABCA1* is expressed in the retinal pigment epithelium, and when ABCA1 protein is inhibited, lipid influx is compromised (Duncan et al., 2009). The poor MPOD response associated with the G allele in our study suggests that these mechanisms may be important: carriers of the G allele not only have lower MPOD but are less able to maintain LZ levels in blood, so might not benefit greatly from LZ supplementation.

We also found an association with a variant rs4926339 (*RPE65*) which was previously linked to MPOD (Meyers et al., 2013). *RPE65* encodes a retinal pigment epithelium protein, which is involved in visual pigment regeneration (Lobo et al., 2012; Xue et al., 2004). A by-product of this cycle is the fluorescent *bis*-retinoid A2E, which has a variety of toxic effects including increase in oxidative damage (Kim et al., 2006; Zhou et al., 2006). Although *RPE65* is a good candidate gene for AMD, there are almost no data on the relationship between *RPE65* and MPOD or L in serum, which makes it difficult to speculate on the mechanism behind our findings. Further functional research is needed before any hypothesis can be drawn.

In this study, rs174534 (*FADS1*) was associated with better MPOD response to supplementation (*p* = 0.03). *FADS1* encodes an enzyme which catalyzes the conversion of plant-derived fatty acids to eicosapentaenoic acid (EPA) (Tanaka et al., 2009). MPOD has been previously associated not only with LZ but also with EPA and docosahexaenoic acid (DHA) (Delyfer et al., 2012). Finally, rs1150561 near *ELOVL2* was associated with poorer L response. The product of this gene catalyzes the elongation of EPA to DHA (Kobayashi et al., 2007). Although our study suggests that *ELOVL2* and *FADS1* polymorphisms may also relate to L and MPOD response respectively, the mechanism of this relationship is uncertain, but it is probably connected to processes governing lipid composition in serum.

Apart from the sample size, a potential limitation of this study is that our sample consisted of Caucasian women only. There is evidence that individuals with African or Asian ancestry have higher MPOD than Caucasian individuals (Wolf-Schnurrbusch et al., 2007; Woo and Lee, 2002) and that there are ethnic differences in AMD prevalence (Friedman et al., 1999; Kawasaki et al., 2010), but it is unclear whether the response to LZ supplementation varies between ethnic groups. Macular pigment density differs between men and women but there are no significant sex differences in plasma LZ concentration or dietary LZ intake (Curran-Celentano et al., 2001; Hammond et al., 1996). There is some evidence of gender differences in magnitude of MPOD response (Thurnham et al., 2008). The prevalence of AMD in men and women is similar, and there is no evidence that the mechanisms of AMD pathogenesis are gender-dependent (Rudnicka et al., 2012). The findings of this study, however, will require validation in other studies including men and individuals from different ethnic backgrounds, though the replication of previous data including men is reassuring. Another point concerning generalizability is whether twins are representative of the general population. The TwinsUK cohort has been shown to be representative of the UK population with respect to physical characteristics and susceptibility to common diseases (Andrew et al., 2001). The study was designed to examine candidate SNPs underlying normal variation, so the subjects included were healthy, without AMD. We believe that studies of endophenotypes such as MPOD are highly relevant to end-stage disease such as AMD.

To summarize, this study is the first to show association between genetic variants and MP response to LZ supplementation and to suggest an important link between processes such as carotenoids transport and lipid metabolism and MP response to supplementation. Further investigation into the pharmacogenomics of LZ supplementation would be worthwhile as it may identify which groups of people are more likely to experience real benefit from taking supplements, as well as explaining why supplement studies have shown considerable variability in supplement response. Understanding the pharmacogenomics of MP supplements may also cast light on the processes involved in macular pigment physiology, and in turn on those involved in AMD pathology.

## Figures and Tables

**Table 1:** Association results for change in lutein and MPOD after supplementation with Lutein and Zeaxanthin.

Gene	SNP	Chr.	Position	MAF	Effect allele	Lutein			MPOD		
						Beta	SE	*p*-value	Beta	SE	*p*-value
***RPE65***	**rs4926339**	**1**	**68694879**	**0.42**	**A**	**−0.04**	**0.016**	**0.01**	**0.029**	**0.014**	**0.04**
*ABCG5*	rs10179921	2	43921795	0.05	A	0.237	0.23	0.3	−0.023	0.028	0.4
***ELOVL2***	**rs1150561**	**6**	**11071833**	**0.02**	**A**	**−0.736**	**0.361**	**0.04**	−0.045	0.053	0.4
***ABCA1***	**rs1929841**	**9**	**106587443**	**0.16**	**G**	−0.177	0.127	0.16	**−0.041**	**0.016**	**0.01**
***FADS1***	**rs174534**	**11**	**61306034**	**0.38**	**G**	−0.001	0.1	0.99	**0.032**	**0.015**	**0.03**
*SCARB1*	rs838879	12	123827394	0.22	G	0.011	0.138	0.94	0.004	0.02	0.84
***SCARB1***	**rs11057841**	**12**	**123882696**	**0.09**	**T**	−0.21	0.245	0.39	**−0.078**	**0.033**	**0.02**
*SCARB1*	rs4379922	12	123917069	0.27	G	0.085	0.109	0.43	0.008	0.013	0.54
*BCMO1*	rs11645428	16	79816397	0.33	A	0.133	0.115	0.25	−0.01	0.014	0.48
*BCMO1*	rs6564851	16	79822098	0.46	G	0.036	0.104	0.73	0.009	0.014	0.51
*BCMO1*	rs6564863	16	79852394	0.43	T	0.04	0.102	0.7	0.001	0.0013	0.96
*ALDH3A2*	rs8069576	17	19510912	0.47	A	−0.041	0.1	0.68	0.006	0.015	0.688

SNP – single nucleotide polymorphism; Chr. – chromosome; Position – physical position of variants is given according to NCBI Human Genome build 36; MAF – minor allele frequency; beta – betas for change in lutein concentration are not back-transformed; SE – standard error; MPOD – macular pigment optical density; bold – SNPs reaching nominal significance.

**Table 2:** Association results for lutein and MPOD at baseline.

Gene	SNP	Chr.	Position	MAF	Effect allele	Lutein			MPOD		
						Beta	SE	*p*-value	Beta	SE	*p*-value
***RPE65***	**rs4926339**	**1**	**68694879**	**0.42**	**A**	−0.079	0.046	0.09	**−0.041**	**0.015**	**0.007**
*ABCG5*	rs10179921	2	43921795	0.05	A	−0.114	0.095	0.23	−0.045	0.031	0.14
*ELOVL2*	rs1150561	6	11071833	0.02	A	0.078	0.164	0.63	−0.003	0.013	0.82
*ABCA1*	rs1929841	9	106587443	0.16	G	−0.02	0.008	0.72	0.004	0.02	0.84
*FADS1*	rs174534	11	61306034	0.38	G	−0.073	0.046	0.11	−0.017	0.015	0.27
*SCARB1*	rs838879	12	123827394	0.22	G	−0.019	0.053	0.72	−0.014	0.018	0.45
***SCARB1***	**rs11057841**	**12**	**123882696**	**0.09**	**T**	**0.252**	**0.173**	**0.01**	0.024	0.035	0.49
*SCARB1*	rs4379922	12	123917069	0.27	G	−0.077	0.046	0.1	−0.023	0.016	0.16
***BCMO1***	**rs11645428**	**16**	**79816397**	**0.33**	**A**	**0.101**	**0.046**	**0.03**	0.025	0.016	0.12
BCMO1*	**rs6564851**	**16**	**79822098**	**0.46**	**G**	**−0.161**	**0.042**	0.0001*	**−0.045**	**0.014**	0.0018*
***BCMO1***	**rs6564863**	**16**	**79852394**	**0.43**	**T**	**−0.038**	**0.015**	**0.01**	**0.03**	**0.014**	**0.04**
*ALDH3A2*	rs8069576	17	19510912	0.47	A	0.048	0.042	0.25	0.016	0.014	0.27

SNP – single nucleotide polymorphism; Chr. – chromosome; Position – physical position of variants is given according to NCBI Human Genome build 36; MAF – minor allele frequency; beta – betas for lutein are not back-transformed; SE – standard error; MPOD – macular pigment optical density; * – significant after Bonferroni correction; bold – SNPs reaching nominal significance.
